# Assessing the risks of capecitabine and its active metabolite 5-fluorouracil to freshwater biota

**DOI:** 10.1007/s11356-023-26505-4

**Published:** 2023-03-30

**Authors:** Cátia Venâncio, Bruna Monteiro, Isabel Lopes, Ana C. A. Sousa

**Affiliations:** 1 Department of Biology, University of Aveiro, Campus de Santiago, P-3810-193 Aveiro, Portugal; 2 CESAM-Centre for Environmental and Marine Studies, University of Aveiro, Aveiro, Portugal; 3 CICECO-Aveiro Institute of Materials, Department of Chemistry, University of Aveiro, Aveiro, Portugal; 4 Department of Biology and Comprehensive Health Research Centre (CHRC), University of Évora, Évora, Portugal

**Keywords:** Combined risk quotient, Anticancer drugs, *Raphidocelis subcapitata*, *Hydra viridissima*, *Danio rerio*

## Abstract

Capecitabine (CAP, prodrug) and 5-fluorouracil (5-FU, its active metabolite) are two of the most prominent cytostatics, for which no clear picture can be drawn regarding potential concentrations of effect for freshwater biota, with CAP being grouped in the least studied cytostatic, whereas 5-FU has been classified as of no and of high environmental risk. Accordingly, the present work aimed to assess the ecotoxicity of CAP and 5-FU in three freshwater species, which included a 72-h assay with the producer *Raphidocelis subcapitata*; a 96-h assay with the invertebrate secondary consumer *Hydra viridissima*; and a 96-h assay with embryos of the vertebrate secondary consumer *Danio rerio*. The following endpoints were monitored: yield and population growth rate for the algae; mortality, morphological alterations, and post-exposure feeding rates for the cnidarian; and mortality, hatching, and malformations for the fish. Overall, organisms’ sensitivity to CAP decreased in the following order: *R. subcapitata* > *H. viridissima* > *D. rerio*, whereas for 5-FU, it decreased in the following order: *H. viridissima* > *D. rerio* > *R. subcapitata*. For CAP, no median lethal effective concentrations (LC/EC_50_) were possible to compute for *D. rerio*, with no significant mortality or malformations registered in embryos exposed at concentrations up to 800 mg L^−1^. For *R. subcapitata*, the EC_50_s were 0.077 and 0.63 mg L^−1^ for yield and growth rate, respectively, and for *H. viridissima*, the EC_50,30 min_ for feeding was 22.0 mg L^−1^. For 5-FU, no EC_50s_ could be computed for *R. subcapitata*, whilst the EC_50s_ for *H. viridissima* mortality and feeding were 55.4 and 67.9 mg L^−1^, respectively, and for *D. rerio*, the LC_50,96 h_ and EC_50,96 h_ (hatching and abnormalities) were 4546, 4100, and 2459 mg L^−1^, respectively. Assuming similar modes of action for both compounds and their co-occurrence, the combined risk quotient of the two chemicals was determined to be 7.97, which represents a risk for freshwater biota. Anticipating the increased consumption of these compounds and cancer development trends worldwide, these impacts may be further aggravated.

**Supplementary Information:**

The online version contains supplementary material available at 10.1007/s11356-023-26505-4.

## Introduction

Contamination of the aquatic environment by cytostatics (or antineoplastic drugs) has been a matter of concern in the last decade due to their widespread occurrence and their potential effects on human and environmental health. With a 70% increase in the incidence rate of cancers predicted in the coming decades (Ferlay et al. 2020; Bray et al. 2021), a concomitant consumption of these compounds is also expected. Note that, for example in Portugal and Spain, the consumption of some cytostatic drugs has already been reported in the order of tonnes per year (Franquet-Griell et al. 2015, 2017; Santos et al. 2017; Cristóvão et al. 2020).

Within the vast list of cytostatic drugs, the prodrug capecitabine (CAP) and its active metabolite 5-fluorouracil (5-FU) are two of the most prominent due to their use in the treatment of various types of cancer (Wishart et al. 2018; Chu and DeVita 2019). CAP was developed as a prodrug of 5-FU, to improve its tolerability and increase patient comfort, as CAP could be administered orally in place of 5-FU intravenous administration (Aguado et al. 2014; Heath et al. 2020). This caused its consumption to shoot up in several countries where it started to be one of the most prescribed cytostatic drugs such as the Czech Republic, France, Germany, India, Italy, the Netherlands, or Spain (Besse et al. 2012; Johnson et al. 2013; Kümmerer et al. 2016; Franquet-Griell et al. 2017; Moermond et al. 2018; Cristóvão et al. 2020).

Cytostatics entrance into the environment is prompted not only by the release of unchanged parent drugs after patient administration, but also by the inefficiency of removal techniques in wastewater treatment plants (Kosjek and Heath 2011; Johnson et al. 2013; Chu and DeVita 2019). Environmental concentrations for both cytostatics are reported to be within the microgram per litre range, with detection levels up to 30 µg L^−1^ and 0.578 µg L^−1^ for CAP and 5-FU, respectively, in surface waters from Japan, Australia, China, and Thailand (Lin et al. 2014; Usawanuwat et al. 2014; Azuma et al. 2015, 2016; Kumar and Pandey 2020). However, some shortcomings have been highlighted that point to expected higher environmental levels of cytostatics. In the case of CAP, this cytostatic has been featured as belonging to the second most prescribed group of cytostatics but occupying the place of one of the least studied ones regarding its toxicity to biota, whereas for 5-FU, some have been pointing that analytical methods are not up-to-date and therefore an environmental risk could not be ruled out (e.g. Tauxe-Wuersch et al. 2006; Gouveia et al. 2019). With expected increased consumption rates, a parallel increase in environmental concentrations is also predicted (namely in the aquatic environments), thus being relevant to report up-to-date environmental values alongside with the assessment of their impacts on non-target organisms at higher concentrations. In the latter case, new ecotoxicity data, enriched with other species and endpoints, must be of priority to enable an accurate risk assessment of these cytostatics. At present, ecotoxicity data available for CAP is very scarce. To our knowledge, the only reported ecotoxicity values for CAP regards biomass and growth rate of the alga *Selenastrum capricornutun* (EC_50,72 h_ of 0.58 mg L^−1^ and 2.0 mg L^−1^), the NOEC for reproduction in *Ceriodaphnia dubia*, and a LC_50,96 h_ of > 867 mg L^−1^ for the fish *Oncorhynchus mykiss* (Straub 2010; Parrella et al. 2014). As for 5-FU, a larger ecotoxicity data set has been published, but some discrepancies exist in the reported effect levels, which introduce uncertainties in conclusions drawn on their potential ecological risk. As an example, the published median effective concentrations, after 72 h of exposure, of 5-FU for *Raphidocelis subcapitata* (following guideline OECD 201, 2011) are as follows: 0.435 mg L^−1^ (Thrupp 2016), 0.13 mg L^−1^ (Brezovšek et al. 2014), and 0.075 mg L^−1^ (Białk-Bielińska et al. 2017), which correspond to almost a sixfold difference between the highest and lowest EC_50,72 h_. Adding to this, most available ecotoxicity data is delivered as NOEC and LOEC (non-observed and lowest observed effective concentrations, respectively), instead of reporting L(E)C_x_ (effect concentrations), which are largely dependent on the range of concentrations and dilution factor used in the ecotoxicity assays. This is very common when assessing the effects of 5-FU on higher trophic levels such as zooplankton species (e.g. *Daphnia* sp., *Ceriodaphnia* sp., or *Brachionus* sp.; Załęska-Radziwiłł et al. 2011; Parrella et al. 2014; Kovács et al. 2016; Białk-Bielińska et al. 2017). Furthermore, it is to highlight that 5-FU was previously considered as of no environmental risk (Straub 2010; Mišík et al. 2019); however, recent updates on its environmental concentrations (hotspot locations) and consumption rates have changed their classification to a compound of high environmental risk (Gouveia et al. 2019), which increases the importance of delivering updated information on aquatic biota for an accurate evaluation.

Within this framework, the present work aimed at thoroughly assessing the ecotoxicity of CAP and 5-FU by generating new data to three freshwater organisms belonging to different taxonomic and functional groups: the microalga *Raphidocelis subcapitata* (a primary producer), the cnidarian *Hydra viridissima*, and the fish *Danio rerio* (both secondary consumers), to allow to compute the risk quotient. Thereunto, several lethal and sub-lethal endpoints were evaluated namely, (1) the yield and growth inhibition of *R. subcapitata*; (2) the mortality, morphological abnormalities, and feeding rate for *H. viridissima*; and (3) the mortality, hatching rate, and percentage of morphological abnormalities for *D. rerio*.

## Materials and methods

### Test solutions

All laboratory procedures were conducted with tight security measures, due to the dangerous properties of these chemicals, and according to the current safety recommendations (Pan American Health Organization 2013; Queruau Lamerie et al. 2013).

Capecitabine (CAP, CAS number 154361–50-9, 98%) and 5-fluorouracil (5-FU, CAS number 51–21-8, 99%) were purchased from Sigma-Aldrich and Fluka, respectively (Table S1). To prepare the stock solutions (Table 1), these cytostatic drugs were dissolved at room temperature under constant stirring in the different culture media of each species (MBL for microalgae, hydra medium, and charcoal-activated filtered tap water for zebrafish) inside a laminar flow chamber. After complete dissolution of the cytostatics, solutions were safely stored at − 15 °C in the dark to minimize any possible degradation and were properly defrosted and diluted in the different culture media to prepare fresh solutions immediately before the beginning of the assays.

**Table 1:** Concentrations of capecitabine (CAP) and 5-fluorouracil (5-FU) used in the ecotoxicity assays

Cytostatic	Species	Culture medium	Stock solution (mg L^−1^)	Concentration range (mg L^−1^)	Dilution factor
CAP	*R. subcapitata*	MBL medium	10.40	0.11–2.09	1.8x
	*H. viridissima*	Hydra medium	1 040	8.19–800	2.5x
	*D. rerio*	Carbon-filtered water	1 000	15.5–800	2.2x
5-FU	*R. subcapitata*	MBL medium	4.0	0.005–0.492	1.5x
	*H. viridissima*	Hydra medium	3 797	50.4–3226	2.0x
	*D. rerio*	Carbon-filtered water	9 537	806–8452	1.6x

### Test species and maintenance

The toxicity of CAP and 5-FU was assessed using freshwater species representative of two functional groups of freshwater ecosystems: the green microalgae *R. subcapitata* (producer), the cnidarian *H. viridissima*, and the fish *D. rerio* (both secondary consumers). These are well-known and studied species recommended/suggested by several guidelines to be used in aquatic toxicity assays (OECD 2011, 2013; Traversetti et al. 2017; Murugadas et al. 2019). The choice of two secondary consumers here — one invertebrate and one vertebrate — was related to potential different exposure pathways. The hydra is characterized by its symbiotic relationship with a green alga, which may influence its response to chemical contamination (e.g. Karntanut and Pascoe 2005), whilst the initial embryonic development of the fish may be protected to some extent by the chorion (e.g. Yang et al. 2020). Both species allowed to evaluate the possible occurrence of teratogenic effects, which are expected effects to occur after exposure to these types of chemicals.

Cultures of *R. subcapitata* were maintained in the laboratory in an aseptic environment and under controlled conditions. The microalgae were cultured in MBL culture medium (Stein 1973) with aeration, at a temperature of 20 ± 2 °C, and a continuous cool-white, fluorescent illumination of – 100 μE m^2^ s^−1^. Prior to the assays, the culture medium and all the material used to prepare the cultures were sterilized in autoclave at 121 °C and 1 Bar, for at least 20 min. Cultures were renewed weekly.

Laboratory cultures of *H. viridissima* were kept in 200-mL glass crystallizers with hydra medium (Trottier et al. 1997), at a controlled temperature of 20 ± 1 °C and a 16:8-h light/dark photoperiod cycle. The cultures were fed ad libitum two times per week, for a period of 30 min in the dark, with a diet of brine shrimp nauplii, obtained from commercially available cysts. After feeding, the organisms were gently washed to eliminate any non-consumed food and were subsequently transferred to a clean medium.

A breeding stock of healthy *D. rerio* fish (wild-type AB) was kept under controlled conditions in a ZebTEC recirculating system (Tecniplast), at the Zebrafish facility at the Department of Biology of the University of Aveiro, Portugal. Fish were maintained in tap water filtered with activated charcoal and reverse osmosis, supplemented with “Instant Ocean Synthetic Sea Salt” (Spectrum Brands, USA). The temperature was maintained at 27 ± 1 °C, conductivity at 794 ± 50 µS/cm, dissolved oxygen equal to or above 95% saturation, a 14:10-h light/dark photoperiod cycle, and the pH was automatically adjusted at 7.5 ± 0.5. Adult fish were fed daily with a commercially available artificial diet Gemma Micro 500 (Skretting®, Spain).

On the day prior to the test, males and females of *D. rerio* were housed in breeding aquaria, where the eggs were deposited in a separate chamber being protected from any possible predation by the adult fishes (Spence et al. 2008). In the morning after, the eggs were collected within 1–2 h after the natural mating, gently rinsed in water from the zebrafish culture system, and inspected under a stereomicroscope (Stereoscopic Zoom Microscope-SMZ 1500, Nikon) (OECD 2013). Unfertilized, coagulated, or injured eggs with obvious irregularities during cleavage were discarded. The remaining eggs were reserved until the assay (5–6 h post-fertilization (hpf)).

### Ecotoxicity assays

#### Growth inhibition assays with *R. subcapitata*

The effects of CAP and 5-FU on the yield and population growth rate of *R. subcapitata* were evaluated according to the OECD standard methodology 201 (OECD 2011), with some minor adaptations to 24-well plates (Moreira-Santos et al. 2004).

Three replicates per concentration (cytostatics diluted in MBL medium) and six replicates for the control group (with MBL medium only) were prepared in 24-well plates (Table 1). To each well was added 1800 μL of the test solution and 200 μL of algal inoculum (3 to 4 days old, at a concentration of 10^5^ cells mL^−1^ to attain a concentration of 10^4^ cells mL^−1^ at the start of the assay). Furthermore, 1 replicate for each treatment (control group and all cytostatics concentrations) was prepared without adding the algae to account for any potential interference of the cytostatics in the absorbance readings. All assays were performed at a controlled temperature of 23 ± 1 °C and under continuous white light at an intensity of 100 μE m^2^ s^−1^. Plates were daily resuspended for a few minutes on an orbital shaker to avoid the settling of the algae and subsequent shadow effects (OECD 2011). Absorbance (*abs*) readings at 440 nm were performed daily (Jenway, 6505 UV/VIS spectrophotometer), and after the subtraction of cytostatics’ *abs* at the same wavelength, the *abs* were converted into cell density per volume (*D*, cells mL^−1^) according to the following Eq. (1) (Venâncio et al. 2017):

1 $$D \left(\mathrm{cells}/\mathrm{mL}\right)= -17107.5+\left(abs \times 7925350\right)$$

Yield (*Y*, biomass produced during the test) was calculated according to Eq. (2), in which *NF* corresponds to the biomass of the algae at the end of the assay (cell mL^−1^) and *NI* to the biomass of the algae at the beginning of the assay (cell mL^−1^):

2 $$Y=NF-NI$$

The percentage of yield inhibition (*Iy*) was determined according to Eq. (3):

3 $$Iy \left(\%\right)=\left(\frac{Yc-Yt}{Yc}\right)\times 100$$

where *Yc* is the mean value for yield in the control group and *Yt* is the value for yield for the cytostatic treatment. The population growth rate (*r*) was evaluated according to Eq. (4):

4 $$r=\frac{\mathrm{ln}NF-\mathrm{ln}NI}{t}$$

in which *NF* is the biomass of the algae at the end of the assay (cell mL^−1^), *NI* is the biomass of the algae at the beginning of the assay, and *t* corresponds to the time of exposure (days). The percentage of growth inhibition (*Ir*) was calculated according to Eq. (5), in which *µC* is the mean growth rate of algae in the control group and *µt* is the growth rate of algae in each cytostatic treatment:

5 $$Ir \left(\%\right)=\left(\frac{\mu C-\mu t}{\mu C}\right)\times 100$$

#### Hydra mortality and morphological assessment assays followed by post-exposure feeding assay

The 96-h acute toxicity assays with *H. viridissima* were performed according to methodologies described by Trottier et al. (1997), adjusted to 24-well plates. Healthy non-budding hydranths were firstly chosen to execute the assays. Six replicates per concentration were set, with a single organism assigned per well, along with 2 mL of hydra medium (control group) or the respective test solution (Table 1). Exposure occurred for 96 h, at 20 ± 1 °C, with a 16:8-h light/dark photoperiod cycle. For the duration of the assay, the organisms were not fed and there was no medium renewal. Mortality and changes in the organisms’ morphology were checked every 24 h under a stereomicroscope, but only the effect after the 96-h exposure period was considered for the estimation of the (sub)lethal concentrations causing 50% of effect [L(E)C_50_]. The scoring of the morphological changes was performed based on the classification of Wilby’s (1988), in which scores ranges from 10 (healthy green hydras) to 0 (dead/disintegrated hydras). Organisms scored with 5 (tulip phase) or lower were considered to be in an irreversible morphological and physiological state and, thus, considered as dead at the end of the assay (Murugadas et al. 2019; Wilby 1988).

At the end of the acute 96-h assays, a 30-min post-exposure feeding assay was carried out with the surviving hydras. For this assay, all surviving hydras (with score ≥ 6) were transferred, individually, to wells with 2 mL of clean hydra medium, and ten brine shrimp nauplii were supplied per individual. Organisms were allowed to feed for 30 min, in total darkness at 20 ± 1 °C (Simões 2015). Afterwards, the remaining brine shrimp in each well were counted and the total number of eaten items was calculated as the subtraction of the initial (*n* = 10) and the final number of brine shrimp.

#### Fish embryo acute toxicity assay with *D. rerio*

Assays with *D. rerio* embryos were performed following the OECD guideline 236 on fish embryo acute toxicity (FET) test (OECD 2013), with small modifications. Thirty eggs per treatment were placed individually in 24-well plates, along with 1 mL of the test solution, plus a control treatment (water from the fish maintenance solely) (Table 1). The assays had a duration of 96 h and were performed under controlled conditions of temperature, at 26 ± 1 °C, and a 16:8 h light/dark photoperiod cycle. Daily observations (Zoom-SMZ 1500 stereomicroscope, Nikon Corporation) included the monitoring of the following apical endpoints: (1) mortality (that included coagulated eggs, arrested development or lack of heartbeat), (2) hatching rate, and (3) phenotypic abnormalities (such as tail and skeletal malformations, oedemas, and delayed development) (Lammer et al. 2009). Cumulative mortality was expressed considering the total number of embryos, whilst cumulative hatching and percentage of organisms with morphological abnormalities were expressed considering the total number of alive embryos.

### Data analysis

Lethal concentrations causing X% of effect (LC_x_) and the respective confidence limits at 95% (CL 95%) were computed through a regression model in Probit software (Sakuma 1998). The estimation of sublethal concentrations resulting in X% of effect (EC_x_) was performed using a non-linear model (three-parametric logistic or sigmoid curve, according to the best fit), resorting to the Statistica for Windows 4.3 software (StatSoft, Aurora, CO, USA).

Prior to statistical analysis, mortality data sets were first Anscombe arcsine transformed, and then, a one-way ANOVA was carried out, followed by Dunnett’s test to determine potential statistical differences against control conditions. Regarding the non-lethal endpoints data sets, normality and homoscedasticity were firstly confirmed with Shapiro–Wilk test and Brown-Forsythe tests, respectively. Afterwards, a one-way ANOVA was carried out followed by Dunnett’s to assess potential differences between treatments and control conditions. Whenever data sets failed one of the assumptions, a non-parametric ANOVA was carried out (Kruskal–Wallis) followed by the multicomparison Dunn’s test. The significance level was set at 0.05. All statistical analysis of variance were processed using the SigmaPlot 14.0 software (Systat Software, Inc. SigmaPlot for Windows).

The risk quotient (*RQ*_*i*_) was computed for both cytostatic drugs by dividing the highest measured environmental concentration (*MEC*) found in the literature and the predicted non-effective concentration (*PNEC*; Eq. 6). An assessment factor of 1000 was applied to the *PNEC* since at least one short-term LC_50_/EC_50_ value from each of three trophic levels was available (Amiard and Amiard-Triquet 2015).

Further calculations for risk quotient determination were performed considering that 5-FU is a metabolite of CAP and similar modes of action are expected as well as their co-occurrence, and thus, additivity in the risk of the two compounds may be assumed. In this sense, the calculation of the risk quotient of their mixture (*RQ*_mix_) was determined by summing the risk calculated for each (Mišík et al. 2019; Eq. 7). The quotient allows the classification of each drug and their mixture from high- to low-risk if *RQ* is higher or lower than 1, respectively.

6 $${\mathrm{RQ}}_{\mathrm{i}}=\mathrm{MEC}/\mathrm{PNEC}$$

7 $${\mathrm{RQ}}_{\mathrm{max}}={\sum }_{i=1}^{n}ROi$$

## Results

### Validity of the assays

Validity criteria required for the different assays, according to the respective guideline and standard protocol, were fulfilled in all experiments (OECD 2011, 2013; Trottier et al. 1997). Accordingly, assays with the microalga *R. subcapitata* resulted in a specific growth of at least 0.92 day^−1^ and the coefficient of variation of specific growth rates did not exceed 7% in the control group (OECD 2011). Concerning the assays with *H. viridissima*, the rate of mortality and percentage of malformations in the control treatment did not exceed 10% at any point of the assay (Trottier et al. 1997). All assays with *D. rerio* also fulfilled the validity criteria regarding the fertilization rate of the eggs (> 70%), temperature of the water inside the wells (26 ± 1 °C), and the overall mortality rate and percentage of morphological abnormalities in the control treatment less than 10% (OECD 2013).

### Toxicity data

**Fig. 1:**
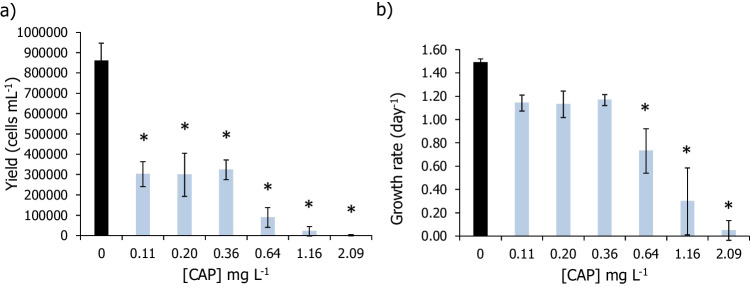
Average of yield (cells mL^−1^) (**a**) and growth rate (day.^−1^) (**b**) of *Raphidocelis subcapitata* after being exposed, for 72 h, to different concentrations of capecitabine (CAP). Vertical bars correspond to the standard deviation. Asterisk indicates a significant statistical difference in relation to control conditions (Dunnett’s or Dunn’s method: *P* < 0.05)

Capecitabine caused significant yield inhibition of *R. subcapitata* at all tested concentrations (Fig. 1a; Dunnett’s method: *P* < 0.001), resulting in an estimated EC_50,72 h_ of 0.077 mg L^−1^, with an 95% CL of 0.025–0.129 mg L^−1^ (Table 2). Concerning its effects on the growth rate of this microalga, CAP caused significant effects at concentrations equal or higher than 0.64 mg L^−1^ (Fig. 1b; Dunn’s method: *P* < 0.050) and, as such, an EC_50,72 h_ (CL 95%) of 0.630 (0.485–0.774) mg L^−1^ was estimated (Table 2).

**Table 2:**
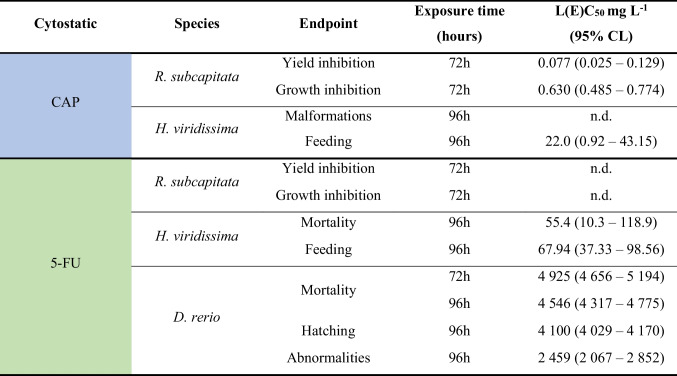
Summary of the concentrations of capecitabine (CAP) and 5-fluorouracil (5-FU), causing 50% of effect (L(E)C_50_), with the 95% confidence limits (95% CL), for the three freshwater model species. n.d., not determined

**Fig. 2:**
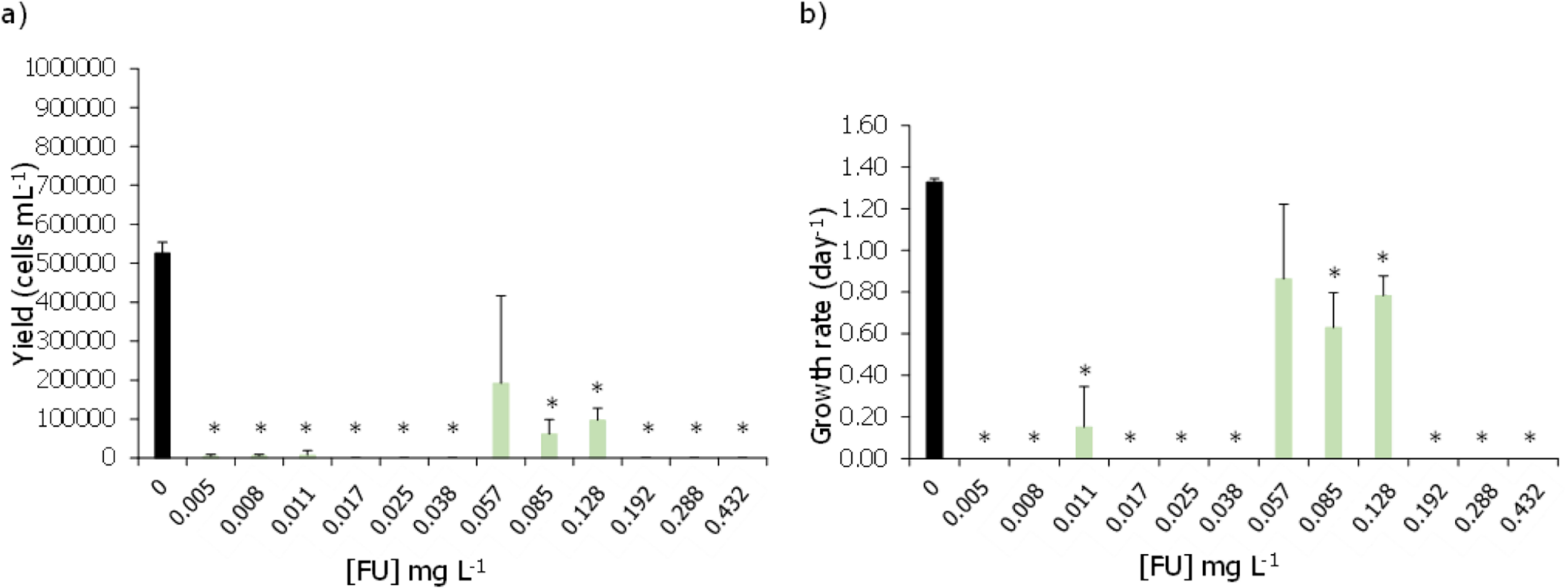
Average of yield (cells mL^−1^) (**a**) and growth rate (day.^−1^) (**b**) of *Raphidocelis subcapitata* after being exposed, for 72 h, to different concentrations of 5-fluorouracil (5-FU). Vertical bars correspond to the standard deviation. Asterisk indicates a significant statistical difference in relation to control conditions (Dunnett’s method: *P* < 0.001)

Regarding the effects of 5-FU on the unicellular green algae *R. subcapitata*, no clear dose–response relation was found (Fig. 2). For yield results, in the lowest concentrations, there was virtually no algal/biomass growth, followed by an increase at the intermediate concentrations and again, at concentrations around 0.057 mg L^−1^, the microalgae yield/growth rate was inhibited (Fig. 2). Even though it was not possible to estimate EC_50_ values, significant differences were observed at all concentrations except at 0.057 mg L^−1^ concerning the effects of 5-FU in the inhibition of both the yield and growth rate of this microalga (Fig. 2; Dunnett’s method: *P* < 0.001).

**Fig. 3:**
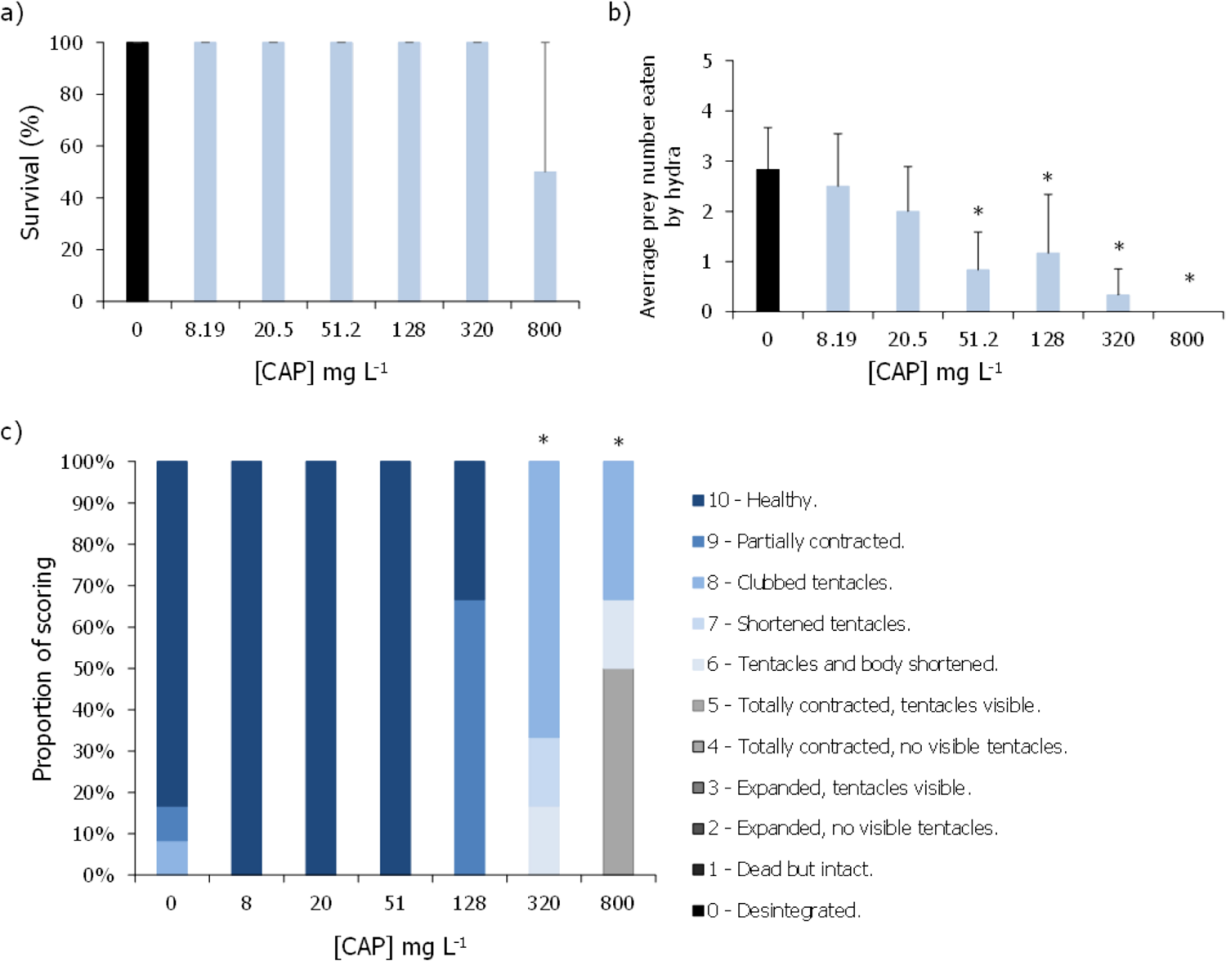
Average survival (%) (**a**), average prey number eaten by organism (**b**), and proportion of morphological scoring (%) based on Wilby’s in 1988 (**c**) of *Hydra viridissima* after being exposed, for 96 h, to different concentrations of capecitabine (CAP). Vertical bars represent the standard deviation. Asterisk indicates a significant statistical difference in relation to control conditions (Dunnett’s or Dunn’s method: *P* < 0.05)

The effects of these two cytostatic drugs on *H. viridissima* were here evaluated for the first time. The prodrug CAP did not significantly affect the survival of these organisms up to the highest tested concentration of 800 mg L^−1^, although at this last concentration 50% of mortality was registered (Fig. 3a). Despite no statistical difference in mortality, concentrations equal to or greater than 51.2 mg L^−1^ caused a significant depression in their feeding rates (Fig. 3b; Dunnett’s method: *P* < 0.05). Moreover, the two highest concentrations of CAP tested (320 and 800 mg L^−1^) also significantly affected the morphological state of the hydras (Fig. 3c; Dunn’s method: *P* < 0.05). Based on the feeding rates obtained results, an EC_50,30 min_ of 22 (0.92–43.15) mg L^−1^ of CAP was computed (Table 2).

**Fig. 4:**
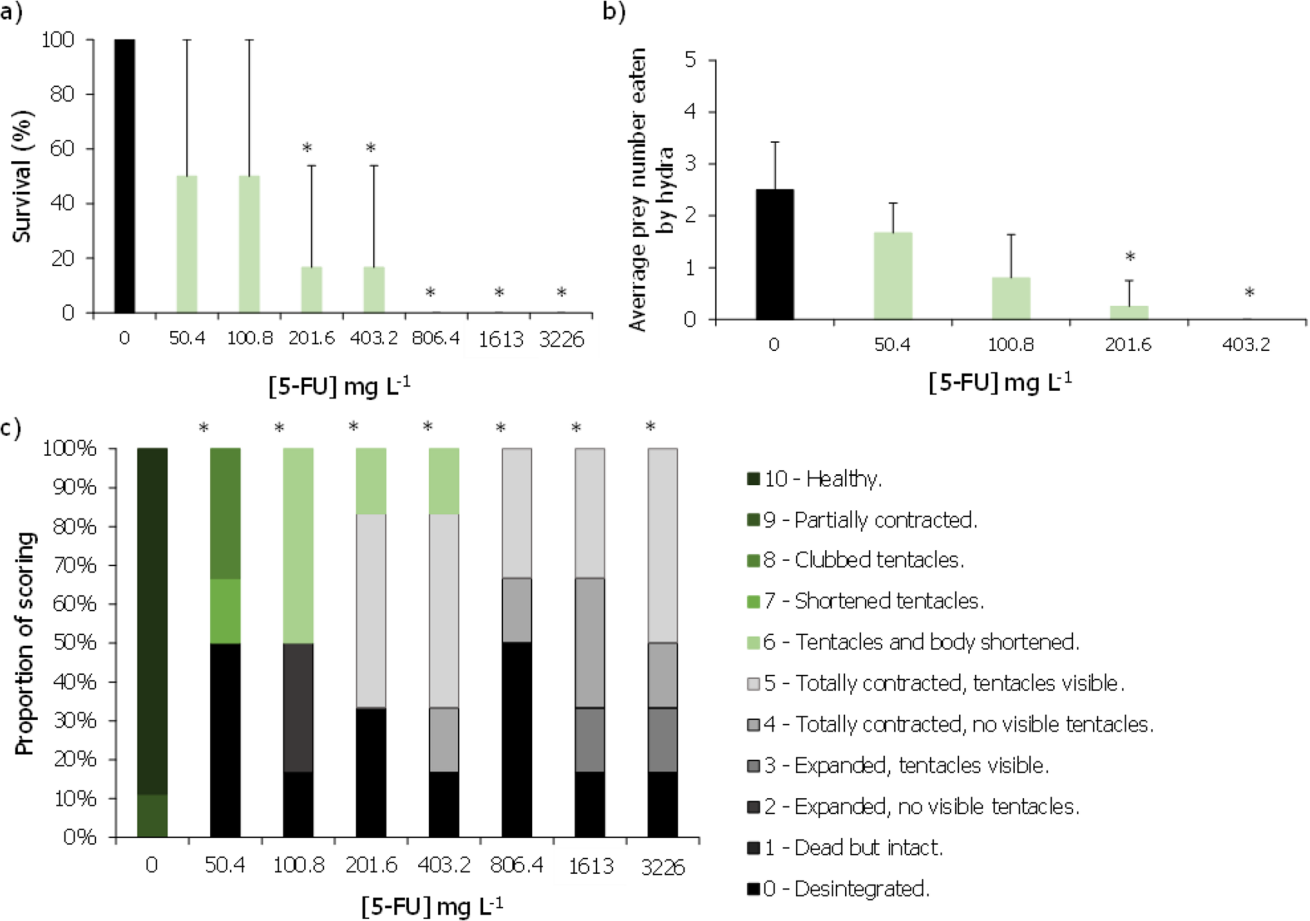
Average survival (%) (**a**), average prey number eaten by organism (**b**), and proportion of scoring (%) based on Wilby’s in 1988 (**c**) of *Hydra viridissima* after being exposed, for 96 h, to different concentrations of 5-fluorouracil (5-FU). Vertical bars represent the standard deviation. Asterisk indicates a significant statistical difference in relation to control conditions (Dunn’s method: *P* < 0.05)

As for 5-FU, this drug caused significant effects for all the evaluated endpoints (Fig. 4). The survival of the hydras was significantly affected at concentrations of 201.6 mg L^−1^ or higher, whereas the morphological state was significantly impacted at all tested concentrations (Fig. 4a, c; Dunn’s method: *P* < 0.05). The computed LC_50,96 h_ was 55.4 (10.3–118.9) mg L^−1^ (Table 2). The feeding rates were significantly decreased at 201.6 and 403.2 mg L^−1^ (Fig. 4b; Dunn’s method: *P* < 0.05). The impacts of 5-FU on the feeding behaviour of these organisms allowed an estimation of a EC_50_ of 67.94 (37.33–98.56) mg L^−1^ (Table 2).

**Fig. 5:**
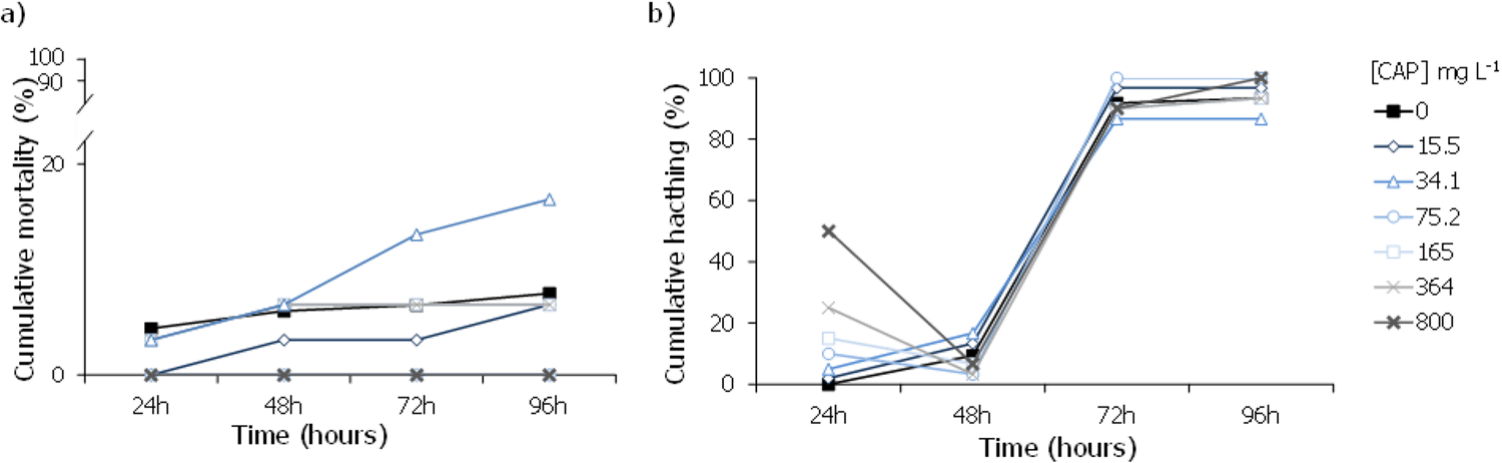
Cumulative mortality (%) (**a**), and cumulative hatching (%) (**b**) caused by the different capecitabine (CAP) concentrations in *Danio rerio* embryos and larvae after being exposed for 24, 48, 72, and/or 96 h

Regarding the assays with zebrafish embryos, exposure to CAP did not result in any significant effects in the survival, percentage of organisms with morphological abnormalities, and hatching rate at all tested concentrations, up to 800 mg L^−1^ (Fig. 5).

**Fig. 6:**
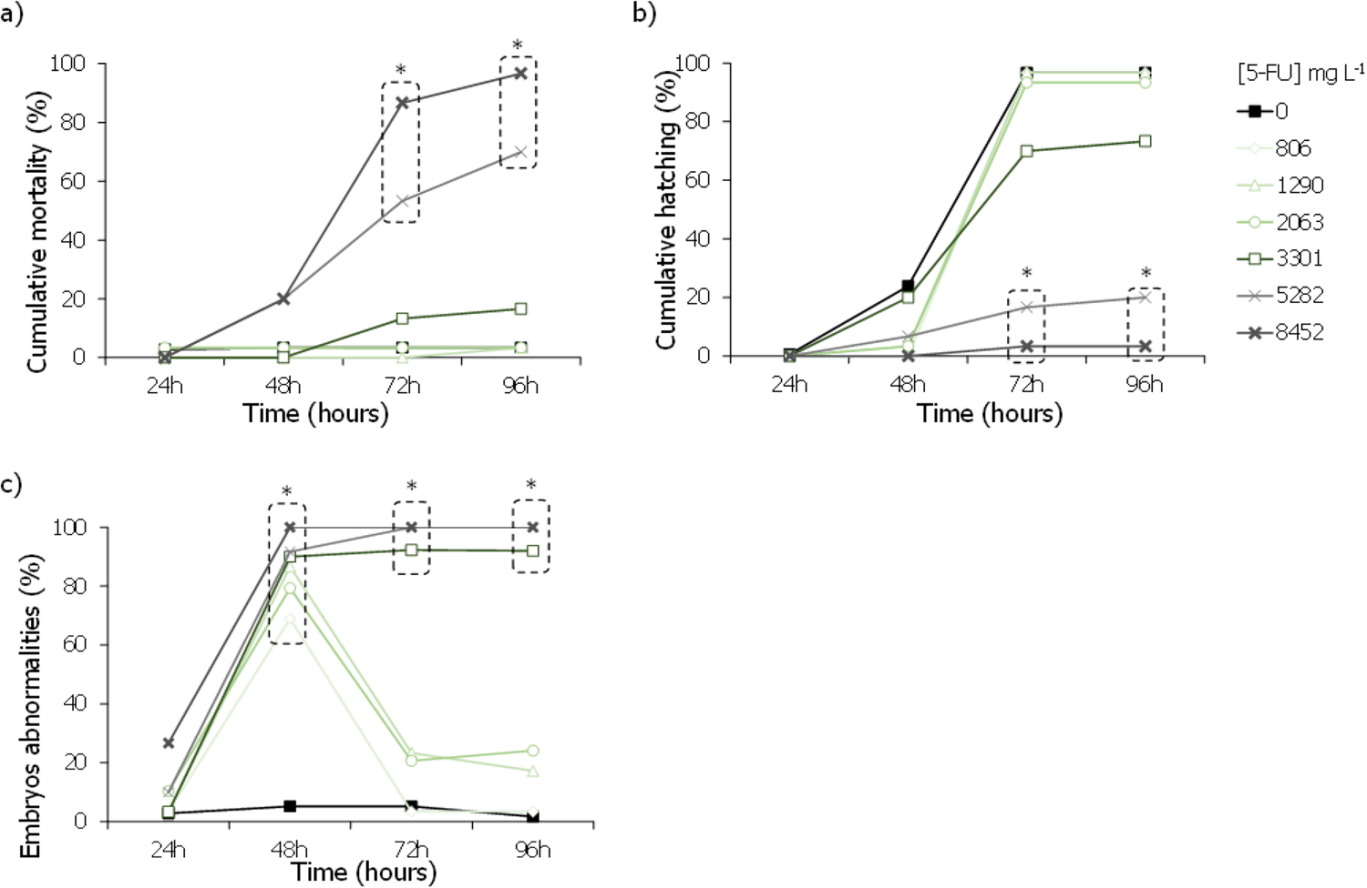
Cumulative mortality (%) (**a**), cumulative hatching (%) (**b**), and percentage of individuals with malformations (%) (**c**) caused by the different 5-fluorouracil (5-FU) concentrations in *Danio rerio* embryos and larvae after being exposed for 24, 48, 72, and/or 96 h. Asterisk indicates a significant statistical difference in relation to control conditions (Dunn’s method: *P* < 0.05). Number sign indicates a highly significant statistical difference concerning the presence of malformations in relation to control conditions (Dunn’s method: *P* < 0.001). The dashed box incorporates all the statistically significant values

In the case of 5-FU, this drug caused significant effects in the survival and hatching rate of *D. rerio* embryos at the two highest concentrations, 5282 and 8452 mg L^−1^, at 72 h of exposure (Fig. 6a, b; Dunn’s method: *P* < 0.001). Accordingly, it was possible to estimate an LC_50,96 h_ value of 4546 (4317–4775) mg L^−1^ and an EC_50,96 h_, for hatching, of 4099.6 (4029.1–4170.1) mg L^−1^, respectively (Table 2).

### Determination of the individual and mixture risk quotient

Concentrations of the cytostatics CAP and 5-FU detected in various aquatic matrices are summarized in Table 3. A brief analysis shows that more values are reported for effluents from healthcare facilities such as hospitals, followed by values reported for effluents from wastewater treatment plants and surface waters (Table 3). As expected, the highest values detected were in hospital effluents; however, especially for 5-FU, some occasionally high values were already reported for the other matrices, such as wastewaters (Mahnik et al. 2007) or surface waters (Usawanuwat et al., 2014).

**Table 3:**
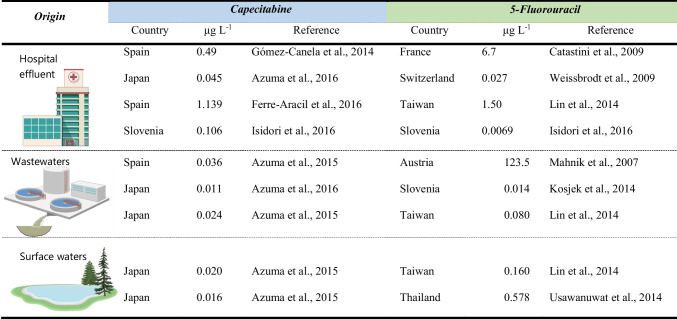
Resume of reported concentrations of both cytostatic drugs on three relevant matrices: hospital effluents, sewage waters after treatment, and surface waters

The risk quotient determination for each compound individually (denoted as *RQ*_*i*_) was determined considering the highest concentration measured in surface waters (*MEC*) of 0.020 and 0.578 µg L^−1^ for CAP and 5-FU (respectively) and the lowest EC_50_/1000 (*PNEC*). Regarding the last parameter, the available studies were collected from the literature (summarized in Table S2) and compared with the data obtained here. The EC_50_ values obtained for the green microalgae *R. subcapitata* in this study were the lowest (0.077 and 0.075 µg L^−1^ for CAP and 5-FU, respectively; Table 2 and Table S2) and, thus, were the ones used to compute the *PNEC* for Cap and 5-FU. The *RQi* derived were of 0.26 and 7.71 for CAP and 5-FU, respectively, meaning that the former presents negligible risk (risk < 1), whilst the later presents risk to the environment (risk > 1). Considering the risk quotient of the mixture (*RQ*_mix_, as one compound is precursor of the other, and may occur and act in biota in a similar way), the value obtained was of 7.97, indicating risk to the environment.

## Discussion

The aim of this study was to provide lethal and sublethal effective concentrations regarding two of the most consumed cytostatic drugs, values which, when combined with updated environmental data, are of paramount importance for an accurate risk evaluation.

In what concerns the effects of CAP in the microalga *R. subcapitata*, the estimated EC_50_ values [EC_50,72 h_ of 0.077 (0.025–0.129) mg L^−1^ and 0.630 (0.485–0.774) mg L^−1^ for yield and growth inhibition, respectively] are two or three orders of magnitude lower than the few ones reported in the literature, namely in a safety data sheet from the ABC Laboratories dating from 1997, which reported an EC_50,72 h_ of 58 mg L^−1^ concerning growth rate inhibition of these microalga, an EC_50,72 h_ of 200 mg L^−1^ for biomass/yield inhibition, and a NOEC value of 14 mg L^−1^ (Hoffmann-La and Ltd 2021). To the authors’ best knowledge, only one other similar toxicity record is described in the literature and it concerns a calculation based on ECOSAR class program, which estimated an EC_50_ of 0.897 mg L^−1^ for green algae, which in turn resulted in the classification of CAP as “very toxic” to these organisms, according to the Globally Harmonized System of Classification and Labelling of Chemicals (Huo et al. 2020).

Based on our results, the effect of 5-FU in the yield and growth inhibition of the microalga did not show a well-defined type of response, subsequently not allowing the estimation of EC_50_ values (Fig. 1; Table 2). Comparing to other similar studies that also assessed the effects of 5-FU in algal species, it does not seem to exist a clear dose–effect curve for this drug, with results available so far including irregular types of sigmoid, non-monotonic, and monotonic non-linear responses (Zounková et al. 2007; Brezovšek et al. 2014). However, contrary to our study, most of these studies were able to estimate EC_50_ values concerning the effects of this drug in the growth of *R. subcapitata*, which ranged between 0.075 and 0.435 mg L^−1^ (Zounková et al. 2007; Załęska-Radziwiłł et al. 2011; Brezovšek et al. 2014; Białk-Bielińska et al. 2017). These inconsistent results amongst studies (with differences in EC_50_ values up to sixfold) may be due to different methodologies followed, i.e., varying volumes per well and/or type of testing apparatus, different guidelines, or presence/absence of constant shaking (Zounková et al. 2007; OECD 2011; Załęska-Radziwiłł et al. 2011; Brezovšek et al. 2014; Białk-Bielińska et al. 2017). Besides these methodological constrains, the toxicity of 5-FU might also be influenced by the ionic strength of the standard medium, which can also explain the large variation in the response to this drug observed in our study (MBL; OECD 2011). Previous studies have reported that even slight changes in the pH (for instance of half unit, that may be a result of the addition of algae at the start of the assay and/or during the assay due to the algae own metabolism) may lead to the formation of different species of the same compound (Markiewicz et al. 2021). In that same study (Markiewicz et al. 2021), the authors studied the acid–base equilibrium of 5-FU along with another cytostatic drug. They found that at a pH closer to 9, dianionic species of 5-FU started to be formed and become predominant from this pH onwards, whilst at lower pH (closer to 7), 5-FU exists mostly in neutral and monoanionic forms (Markiewicz et al. 2021). Despite that it has been argued before that dianionic forms of 5-FU are strongly limited in aqueous media (Wielińska et al. 2019), one cannot say for sure if the presence, even in smaller amounts of these other forms, may be more or less toxic to aquatic biota as no evidence has been provided so far in the literature. These conflicting ecotoxicity data reported for 5-FU highlight the need to uniformize testing procedures in order to reduce the uncertainties associated with the risk assessment of this cytostatic.

In the present work, the effects of CAP and 5-FU are described for the first time for *H. viridissima*, having both cytostatics proved to induce toxic effects to this organism. Whilst CAP significantly affected the morphological state and feeding behaviour of these organisms at concentrations ranging from 51.2 to 800 and 320 to 800 mg L^−1^, respectively (Fig. 3) but with no significant mortality, its active metabolite 5-FU significantly affected all the studied endpoints, with significant morphological alterations being registered at the lowest tested concentration of 50.1 mg L^−1^ (Fig. 4). Given that 5-FU is the active metabolite of CAP, and thus responsible for its pharmaceutically active properties, it is not surprising that 5-FU could cause significant effects on the survival and condition of these organisms at concentrations relatively lower than those observed for CAP. The sensitivity of *Hydra* sp. stood out in cases in which other groups failed to deliver the desired benchmark levels for risk assessment. For instance, for CAP, no effective concentrations could be delivered when assessing its effects on zebrafish, whilst the same happens in 5-FU with the green microalgae. Despite that, the sublethal endpoints tested for *H. viridissima* were sensitive and informative of potential cellular, individual, and population disruptive levels of these cytostatics (Lee et al. 2020).

Regarding the assessment of the impact of these drugs on zebrafish, CAP did not result in any significant effects on *D. rerio* embryos and larvae up to the highest tested concentration of 800 mg L^−1^ (Fig. 5). To the authors’ best knowledge, the only toxicity value available in the literature regarding the effect of this drug in fish species, namely for *O. mykiss,* is described in the safety data sheet from Roche, with an estimated no-observable effect concentration (NOEC) value higher than 867 mg L^−1^ (Hoffmann-La and Ltd 2021). This absence of studies does not come as a surprise when considering that CAP is the prodrug of 5-FU, the latter being the pharmacologically active form.

Regarding the assays with 5-FU, this drug caused significant effects on the survival, hatching, and development of malformations on these organisms (Fig. 6). It was possible to estimate an LC_50,96 h_ value of 4546 mg L^−1^ and an EC_50,96 h_ regarding impacts on the hatching rate of 4099.6 mg L^−1^ (Table 2). These values are higher than the few ones reported in the literature for *D. rerio*, namely in the study by Kovács et al. (2016), which reported an LC_50,96 h_ of 2610 mg L^−1^, and other authors were only able to estimate LC_50_ values to be higher than at least 100 mg L^−1^ (Załęska-Radziwiłł et al. 2011; Klein et al. 2021). A similar pattern has also been observed for other fish species, like *Lebistes reticulatus*, *Pimephales promelas*, or *O. mykiss*, with reports of toxicity values higher than 100 mg L^−1^, up to 2420 mg L^−1^ (DeYoung et al. 1996; Załęska-Radziwiłł et al. 2011). Most of the studies, which were not able to determine definite ecotoxicological values, did not test such higher concentrations of 5-FU, as the ones assessed in our study (Załęska-Radziwiłł et al. 2011; Klein et al. 2021).

Calculating the risk quotient of the cytostatics individually showed that CAP present no environmental risk, whereas the same was not true for 5-FU. Gouveia et al. (2019) also reported that CAP does not present a risk for freshwater biota. Though, these same authors reported 5-FU as of high environmental concern (Gouveia et al. 2019), estimating a *RQ* much higher than that of the present study (963 *cfr*. 7.97), which is related to the calculation method. In the present study, the *RQ* was estimated using short-term effective concentrations, whereas Gouveia et al. (2019) based their *RQ* estimation on long-term NOECs (applying a lower AF = 10), which is highly dependent on the range of concentrations tested in each experiment that was carried out.

If one considers that both compounds have similar modes of action (as one is the pro-drug of the other) and their co-occurrence in the environment, it is expected that the derivation of the combined risk is a more realistic and reliable scenario than the *RQ* estimation for each individually. *RQ*_mix_ proved that there was an environmental risk. Although the estimated effective values were much higher than those found in environmental matrices, this type of study is still fundamental as it may help direct scientific research into the development of pro-drugs and their fate to reduce the risks associated with the environment. Of the three organisms tested, microalgae (producer), hydra, and zebrafish (secondary consumers), CAP was always less toxic than 5-FU. For example, for the cnidarian morphological state, there was a difference of two orders of magnitude between both cytostatics, whilst for the fish, this difference is even more pronounced with no significant mortality or malformations caused by CAP against the L(E)C_x_ in the thousand orders of milligrams regarding 5-FU (Figs. 5 and 6). These results may be explained by the fact that CAP is a pro-drug of 5-FU, with different modes of administration and of metabolization. Following administration, CAP is adsorbed in the gastrointestinal mucosa and suffers a 3-step enzymatic conversion resulting in 5-FU: briefly, (1) in the liver, CAP is metabolised by hepatic carboxylesterase to 5′-deoxy-5-fluorocytidine (5′-DFCR), (2) which is then converted in 5′-deoxy-5-fluorouridine (5′-DFUR) by cytidine deaminase, and finally, (3) thymidine phosphorylase hydrolyses 5′-DFUR to 5-FU, the active metabolite (Chu and DeVita 2019). This last enzyme is found in both normal and tumour tissues, albeit it is expressed at higher levels in the latter. Such factor renders capecitabine a higher tumour-targeting specificity, which in turn justifies the overall lower systemic toxicity and adverse side effects (Roche Pharma AG, no date), thus supporting the lower toxicity here presented towards the secondary consumers (with no significant morphological abnormalities were observed in *D. rerio*, for example). Subsequently, or in the case of 5-FU administration, this drug is metabolized to three active metabolites, namely 5-fluoro-2-deoxyuridine monophosphate (FdUMP), fluorodeoxyuridine triphosphate (FdUTP), and 5-fluorouridine triphosphate (FUTP), and one inactive metabolite, dihydrofluorouracil (Chu and DeVita 2019, Roche Pharma AG, no date). Afterwards, this set of products can cause injury to healthy and non-healthy cells by two different mechanisms/methods. On one hand, during the synthesis of RNA, FUTP, one of the active metabolites of 5-FU, can be mistakenly incorporated in place of uridine triphosphate (UTP) by nuclear transcriptional enzymes. This error may disrupt RNA processing, mRNA translation, and protein synthesis (Chu and DeVita 2019, Roche Pharma AG, no date). In contrast, the metabolite FdUMP can bind to thymidylate synthase, inhibiting the formation of thymidylate — a precursor of thymidine triphosphate — which in turn is crucial for the synthesis of DNA. These metabolic alterations corroborate the high malformations percentage found both in the cnidarian and the fish, as well as the high potential to cause damage in non-target species once reaching the environment as previously hypothesized.

A brief look at the sensitivity of the species studied highlighted the importance of toxicity assays with organisms from which it is possible to assess other endpoints besides the usual lethality data. Notwithstanding, both secondary consumers used in this study, allowed the observation of morphological malformations which may be indicative of potential teratogenic effects and disruption of the normal cellular cycle. The evaluation of these events/consequences are of crucial importance namely for the risk assessment of this type of drugs given its known mutagenic, genotoxic, and carcinogenic properties. Accordingly, the freshwater cnidarian *H. viridissima* proved to be a very useful species to account for the effects of these type of compounds since they indicated effects which were not observed or computed for other (standard) species (e.g. *R. subcapitata* for 5-FU or *D. rerio* for CAP).

## Conclusions

The anticipated increase in the use of cytostatic drugs in the upcoming years stresses the need for a comprehensive assessment of the toxicity of these drugs towards aquatic organisms. Herein, we describe the ecotoxicological profile of the prodrug capecitabine and its active metabolite 5-fluorouracil towards three freshwater species representative of two trophic levels, namely the algae *R. subcapitata,* and the secondary consumers *H. viridissima* and *D. rerio.* The results here described provide, for the first time, toxicity data for the cnidarian *H. viridissima* and reinforces the usefulness of this organism in ecotoxicological studies.

As expected, the active metabolite 5-FU tended to exhibit higher toxicity to the tested organisms, with lethal and effective concentrations two to three orders of magnitude apart between CAP and 5-FU. When comparing the toxicity profiles of the drugs between the different test species, CAP induced only sublethal effects on *R. subcapitata* and *H. viridissima*, posing higher toxicity to the microalgae. On the other hand, 5-FU induced both lethal and sublethal effects on *H. viridissima* and *D. rerio*, with cnidarians being more sensitive than the fish. Furthermore, though a negligible risk was computed for CAP, the *RQ* values for 5-FU and its mixture with CAP revealed an existing ecological risk. These results suggest that it is important not only to compare parent compounds and pro-drugs, but also to focus on the integration of several trophic levels and endpoints. By providing such data, it will be possible to derive more integrative conclusions regarding the environmental hazards posed by these drugs.

## Supplementary Information

Below is the link to the electronic supplementary material.

Supplementary file1 (DOCX 44.4 KB)

## Data Availability

The data sets generated during and/or analysed during the current study are available from the corresponding author on reasonable request.
