# Supplementary material for: Assessing the risks of capecitabine and its active metabolite 5-fluorouracil to freshwater biota
Source: Environ Sci Pollut Res Int. 2023 Mar 30;30(20):58841–54. doi: 10.1007/s11356-023-26505-4 (PMC10163094; doi:10.1007/s11356-023-26505-4)
Supplement: Supplementary file 1 — Supplementary file1 (DOCX 44.4 KB) [file 11356_2023_26505_MOESM1_ESM.docx]

**Supplementary material**

**Table S1:** Name, molecular and structural formulas, and chemical and physical properties of the two studied cytostatics. Data extracted from (National Center for Biotechnology Information, 2021a, 2021b).

|  | Capecitabine | 5-Fluorouracil |
| --- | --- | --- |
| Chemical formula  Molecular weight  Water solubility  Partition Coefficient  Acid dissociation constant | C_15_H_22_FN_3_O_6_  M.w. = 359.35 g mol^-1^  S_H2O_ = 26 000 mg L^-1^  LogP = 0.4  pKa = 1.9 (amide) | C_4_H_3_FN_2_O_2_  M.w. = 130.08 g mol^-1^  S_H2O_ = 11 100 mg L^-1^  LogP = -0.89  pKa = 8.02 |
| Structural formula | 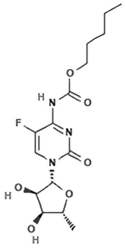 | 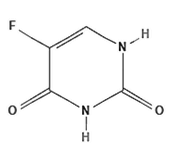 |

National Center for Biotechnology Information. (2021a). PubChem Compound Summary for CID 3385, 5-Fluorouracil. <https://pubchem.ncbi.nlm.nih.gov/compound/5-Fluorouracil>

National Center for Biotechnology Information. (2021b). PubChem Compound Summary for CID 60953, Capecitabine. <https://pubchem.ncbi.nlm.nih.gov/compound/Capecitabine>

**Table S2:** Summary of effective concentrations reported for freshwater biota exposed to the two cytostatic drugs, 5-Fluorouracil and Capecitabine. NOEC – non-observed effective concentration. LOEC – lowest observed effective concentration. L(E)C_50_ – median lethal or effective concentration.

|  |  | **Capecitabine** | | |  | **5-Fluorouracil** | | |
| --- | --- | --- | --- | --- | --- | --- | --- | --- |
| **Species** |  | **NOEC** mg/L | **LOEC** mg/L | **L(E)C_50_** mg/L |  | **NOEC** mg/L | **LOEC** mg/L | **L(E)C_50_** mg/L |
| *Vibrio fischeri* |  |  |  | **2.16**  Luminescence  Barışçı et al., 2018 |  |  |  | **0.12**  Luminescence  Backhaus et al., 2000 |
| *Tetrahymena thermophila* |  |  |  |  |  | **0.195**  Growth, 24h  Zaleska et al., 2011 |  | **44.02**  Growth, 24h  Zaleska et al., 2011 |
| *Anabaena flos-aquae* |  |  |  |  |  | **0.002**  Growth, 72h  Straub et al., 2010 |  |  |
| *Pseudokerchneriella subcapitata* |  | **0.14**  Growth, 72h  Straub et al., 2010 |  |  |  | **0.01**  Growth, 72h  Misík et al., 2019 |  |  |
| *Raphidocelis subcapitata* |  |  | **0.11**  Yield, 72h  **0.62**  Growth, 72h  **This study** | **0.077** (0.025 – 0.129)  Yield, 72h  **0.630** (0.485 – 0.774)  Growth, 72h  **This study** |  |  | **0.005**  Yield, 72h  **0.005**  Growth, 72h  **This study** | **0.075**  Growth, 72h  Bialk-Bielinska et al., 2017 |
| *Synechococcus leopoliensis* |  |  |  |  |  | **0.12**  Growth, 72h  Misík et al., 2019 | **0.39**  Growth, 72h  Brezovšek et al., 2014 | **1.20**  Growth, 72h  Brezovšek et al., 2014 |
| *Lemna minor* |  |  |  |  |  |  |  | **2.45**  Growth, 7d  Bialk-Bielinska et al., 2017 |
| *Brachionus calyciflorus* |  | **3.12**  Reproduction, 48h  Parella et al., 2014 | **6.25**  Reproduction, 48h  Parella et al., 2014 | **15.4**  Reproduction, 48h  Parella et al., 2014 |  | **0.125**  Reproduction, 48h  Parella et al., 2014 | **0.250**  Reproduction, 48h  Parella et al., 2014 | **0.322**  Reproduction, 48h  Parella et al., 2014 |
| *Ceriodaphnia dubia* |  | **0.6**  Reproduction, 7d  Parella et al., 2014 | **1.9**  Reproduction, 7d  Parella et al., 2014 | **2.4**  Reproduction, 7d  Parella et al., 2014 |  | **0.00222**  Reproduction, 7d  Misík et al., 2019 |  | **501**  Mortality, 24h  Parella et al., 2014 |
| *Daphnia magna* |  | **1.9**  Reproduction, 21d  Parella et al., 2014 | **6.1**  Reproduction, 21d  Parella et al., 2014 | **224**  Mortality,24h  **20.5**  Reproduction,21d  Parella et al., 2014 |  | **0.00206**  Reproduction, 21d  Misík et al., 2019  **0.00006**  Reproduction,21d | **0.05**  Reproduction,21d  Zounková et al., 2010 | **20.84**  Mortality, 48h,  Parella et al., 2014  **0.0015**  Reproduction,28d |
|  |  |  |  |  |  | Załęska-Radziwiłł et al, 2011 |  | Załęska-Radziwiłł et al, 2011 |
| *Thamnocephalus platyurus* |  |  |  | **197.7**  Mortality, 24h  Parella et al., 2014 |  |  |  | **0.28**  Mortality, 24h  Parella et al., 2014 |
| *Hydra viridissima* |  | **20.5**  Feeding, 30 min  **128**  Malformations, 96h  **This study** | **51.2**  Feeding, 30 min  **320**  Malformations, 96h  **This study** | **22.0**  Feeding, 30 min  **This study** |  | **100.8**  Mortality, 96h  **100.8**  Feeding, 30 min  **This study** | **201.6**  Mortality, 96h  **201.6**  Feeding, 30 min  **50.4**  Malformations, 96h  **This study** | **55.4**  Mortality, 96h  **67.9**  Feeding, 30 min  **This study** |
| *Danio rerio* |  |  |  |  |  | **1.0**  Mortality, 33 days  Misík et al., 2019  **3301**  Mortality, 96h  **This study**  **3301**  Hatching, 96h  **This study**  **2063**  Malformations, 96h  **This study** | **5282**  Mortality, 96h  **This study**  **5282**  Hatching, 96h  **This study**  **3301**  Malformations, 96h  **This study** | **2610**  Mortality, 96h  **1723**  Malformations, 120h  Kovacs et al., 2016  **4100**  Hatching, 96h  **This study** |
| *Pimephales promelas* |  |  |  |  |  |  | **20**  Growth, 120h  DeYoung et al., 1996 |  |
| *Xenopus laevis* |  |  | **20**  Malformations, 96h  Isidori et al., 2016 |  |  |  | **50**  Malformations, 96h  Isidori et al., 2016 |  |

**Reference**

Backhaus T, Altenburger R, Boedeker W, Faust M, Scholze M, Grimme LH (2000) Predictability of the toxicity of a multiple mixture of dissimilarly acting chemicals to Vibrio fischeri. Environ Toxicol Chem: an International Journal 19(9):2348–2356.
